# Association Between Historical Neighborhood Redlining and Cardiovascular Outcomes Among US Veterans With Atherosclerotic Cardiovascular Diseases

**DOI:** 10.1001/jamanetworkopen.2023.22727

**Published:** 2023-07-11

**Authors:** Salil V. Deo, Issam Motairek, Khurram Nasir, Amgad Mentias, Yakov Elgudin, Salim S. Virani, Sanjay Rajagopalan, Sadeer Al-Kindi

**Affiliations:** 1 Surgical Services, Louis Stokes Veteran Affairs Hospital, Cleveland, Ohio; 2 Harrington Heart and Vascular Institute, University Hospitals, Cleveland, Ohio; 3 Houston Methodist Hospital, Houston, Texas; 4 Heart and Vascular Institute, Cleveland Clinic, Cleveland, Ohio; 5 Michael E. DeBakey Veterans Affairs Medical Center, Section of Cardiovascular Research, Baylor College of Medicine, Houston, Texas

## Abstract

**Importance:**

In the 1930s, the government-sponsored Home Owners’ Loan Corporation (HOLC) established maps of US neighborhoods that identified mortgage risk (grade A [green] characterizing lowest-risk neighborhoods in the US through mechanisms that transcend traditional risk factors to grade D [red] characterizing highest risk). This practice led to disinvestments and segregation in neighborhoods considered redlined. Very few studies have targeted whether there is an association between redlining and cardiovascular disease.

**Objective:**

To evaluate whether redlining is associated with adverse cardiovascular outcomes in US veterans.

**Design, Setting, and Participants:**

In this longitudinal cohort study, US veterans were followed up (January 1, 2016, to December 31, 2019) for a median of 4 years. Data, including self-reported race and ethnicity, were obtained from Veterans Affairs medical centers across the US on individuals receiving care for established atherosclerotic disease (coronary artery disease, peripheral vascular disease, or stroke). Data analysis was performed in June 2022.

**Exposure:**

Home Owners’ Loan Corporation grade of the census tracts of residence.

**Main Outcomes and Measures:**

The first occurrence of major adverse cardiovascular events (MACE), comprising myocardial infarction, stroke, major adverse extremity events, and all-cause mortality. The adjusted association between HOLC grade and adverse outcomes was measured using Cox proportional hazards regression. Competing risks were used to model individual nonfatal components of MACE.

**Results:**

Of 79 997 patients (mean [SD] age, 74.46 [10.16] years, female, 2.9%; White, 55.7%; Black, 37.3%; and Hispanic, 5.4%), a total of 7% of the individuals resided in HOLC grade A neighborhoods, 20% in B neighborhoods, 42% in C neighborhoods, and 31% in D neighborhoods. Compared with grade A neighborhoods, patients residing in HOLC grade D (redlined) neighborhoods were more likely to be Black or Hispanic with a higher prevalence of diabetes, heart failure, and chronic kidney disease. There were no associations between HOLC and MACE in unadjusted models. After adjustment for demographic factors, compared with grade A neighborhoods, those residing in redlined neighborhoods had an increased risk of MACE (hazard ratio [HR], 1.139; 95% CI, 1.083-1.198; *P* < .001) and all-cause mortality (HR, 1.129; 95% CI, 1.072-1.190; *P* < .001). Similarly, veterans residing in redlined neighborhoods had a higher risk of myocardial infarction (HR, 1.148; 95% CI, 1.011-1.303; *P* < .001) but not stroke (HR, 0.889; 95% CI, 0.584-1.353; *P* = .58). Hazard ratios were smaller, but remained significant, after adjustment for risk factors and social vulnerability.

**Conclusions and Relevance:**

In this cohort study of US veterans, the findings suggest that those with atherosclerotic cardiovascular disease who reside in historically redlined neighborhoods continue to have a higher prevalence of traditional cardiovascular risk factors and higher cardiovascular risk. Even close to a century after this practice was discontinued, redlining appears to still be adversely associated with adverse cardiovascular events.

## Key Points

**Question:**

Is historical redlining associated with cardiovascular disease outcomes in US veterans?

**Findings:**

In this cohort study, 79 997 veterans residing in redlined areas (Home Owners’ Loan Corporation–established maps of US neighborhoods considered high risk for mortgage) had a higher risk of major adverse cardiovascular events and all-cause mortality compared with individuals in areas considered not at high risk. This association was also noted independent of traditional cardiovascular risk factors.

**Meaning:**

The findings of this cohort study suggest that redlining is still adversely associated with cardiovascular events throughout the US.

## Teaser

This cohort study compares adverse cardiovascular events in US veterans residing in high-risk neighborhoods, as categorized by the Home Owners’ Loan Corporation, with those in residents of neighborhoods of lower risk.

## Introduction

The Home Owners’ Loan Corporation (HOLC), created in the 1930s to increase homeownership for working-class US individuals under the New Deal, established a color-coded grading system for neighborhoods in more than 200 US cities according to foreclosure risk.^1^ The ratings of these areas were largely based on racial and ethnic compositions and would inform lending risk. Neighborhoods were rated and thus color-coded as A (best [green]), B (still desirable [blue]), C (definitely declining [yellow]), and D (hazardous [red]). This practice, later termed neighborhood redlining, led to decades of disinvestments in redlined neighborhoods and exacerbated residential segregation. This designation ultimately resulted in profound consequences as a direct sequalae of racist policies, culminating in worse neighborhood-level health risk factors, access to care, and adverse environmental exposures in redlined areas.^2^

Color coding was discontinued in the 1940s. However, it is still unclear whether living in these neighborhoods continues to have a negative association with cardiovascular outcomes. Although prior studies have consistently shown worse health outcomes in redlined areas, these reports have limitations, including population-level data, small sample size, and ecological study design, using neighborhoods as the unit of analysis.^3,4^ Additionally, it is unclear to what extent these data can be extrapolated as these historical neighborhoods experience marked flux owing to instability due to socioeconomic conditions. To fill these knowledge gaps, we analyzed patient-level data from a national cohort of US veterans to evaluate whether this historical practice continues to be associated with adverse cardiovascular outcomes.

## Methods

### Overview of the Study Cohort

The study was approved by the Louis Stokes Cleveland VHA Medical Center Institutional Review Board and the requirement for individual patient consent was waived given no/minimal risk to patients (only data breach). This report follows the Strengthening the Reporting of Observational Studies in Epidemiology (STROBE) reporting guideline for cohort studies. This retrospective cohort study with individual patient linkage was performed using electronic health records and pharmacy, laboratory, geospatial, and vital status information stored in the Veterans Affairs (VA) National Computing infrastructure, which includes VA and non-VA claims data. Using the *International Statistical Classification of Diseases and Related Health Problems, 10th Revision, Clinical Modification* codes, we first identified patients (1 113 607 visits; 784 096 patients) with a primary diagnosis of stable atherosclerotic vascular disease (coronary artery disease, peripheral artery disease, or cerebrovascular disease) who received outpatient care (January 1, 2016, to December 31, 2019) with their first visit defined as the index visit. Data analysis was performed in June 2022.

We obtained patients’ census tract information from self-reported residential addresses during their index visit. We excluded 704 099 patients who resided outside the HOLC map coverage areas.

### Exposure Assessment

We used HOLC data from the Mapping Inequality Project digitalized maps.^5^ Geographic areas graded by the HOLC have different geographic boundaries than current geographic divisions by the Census Bureau. As described previously,^3,6,7,8^ we generated a census tract–based HOLC grading, weighted according to the area intersection with previously redlined neighborhoods.

First, using open-source geographic information system software (QGIS version 3.16), we quantified percentage intersections between current US census tract boundaries and HOLC-graded neighborhoods. We excluded census tracts having less than 20% total intersection with HOLC-graded neighborhoods, as not substantially affected by redlining. For each census tract, the intersection with an HOLC neighborhood was divided by the total intersections for that census tract. We then multiplied these fractions by the HOLC numeric score equivalents (HOLC scores 1-4 corresponding to HOLC grades A-D) to generate an HOLC continuous score for each area of intersection. The HOLC scores for areas of intersections were summed for each census to generate an HOLC score for each census tract that was then rounded and transformed into 1 of the 4 HOLC grades: A (1), B (2), C (3), and D (4). For example, if 25% of the census tract’s intersection was graded as D (numeric score 4) and 75% was graded as A (numeric score 1), then the census tract numeric score would be (0.25 × 4 + 0.75 × 1 = 1.75). This score was then rounded and transformed back into 1 of 4 categories: A (1), B (2), C (3), and D (4) corresponding to the historical HOLC grades. For example, 1.75 would round up to 2, corresponding to HOLC grade B. Redlined neighborhoods were defined as D-graded neighborhoods. Each veteran in the cohort was assigned the HOLC grade of the census tract of residence at the time of visit. We ultimately generated approximately 13 480 HOLC census tracts (approximately 16% of total US census tracts).

We additionally performed an analysis that included all patients using a continuous HOLC score. To derive a score for each census tract in the US, we initially determined the proportion of the tract’s land area that had been rated by the HOLC. Subsequently, we multiplied the proportion of the tract’s area that intersected with each HOLC grade by the corresponding numerical value, ranging from 1 (A) to 4 (D). Areas within census tracts that did not intersect with HOLC-graded areas were allocated a value of 0. For example, in a hypothetical census tract with 50% of the area graded B and the other 50% without intersection with HOLC-graded areas, the score would be calculated as ([2 × 0.50] + [0] = 1). Tracts with no intersection with HOLC-graded areas were assigned a score of 0 (100% × 0 = 0). This approach enables calculation of a score for every census tract in the US, regardless of percent intersections with HOLC-graded areas.

### Identification of Covariates

Patient-level covariates, namely, age at index visit, sex, self-reported race (American Indian or Alaska Native, Asian, Black, White, declined to report, and unknown) and ethnicity (Hispanic, Not Hispanic, and unknown), presence of diabetes, hypertension, chronic kidney disease, atrial fibrillation, smoking status, and history of myocardial infarction (MI) or percutaneous coronary intervention, were identified from inpatient and outpatient data up to 1 year before their index visit. Race and ethnicity were included in the model to evaluate whether redlining association with MACE was independent of race and ethnicity. Census tract–derived median household income for each patient was obtained using the US Department of Housing and Urban Development data, US Census Bureau data, and the patients’ residential address.

The community deprivation index is often observed as an important indicator of health outcomes. Therefore, a well-validated community deprivation index score was obtained for each patient based on their residential census tract. This community deprivation index, measured on a continuous scale, is derived using the following 6 census-tract measures: fraction of the population below the poverty level, median household income, fraction of the population with at least a high-school education, fraction of the population without health insurance, fraction of the population receiving public assistance income, and fraction of vacant houses in that census tract. The single summary score is then rescaled to fit between 0 (least) and 1 (most deprived).^9^ Based on their rescaled community deprivation index scores, patients were then grouped into the following tertiles of deprivation: I (least), II (moderate), and III (most).

### End Points

We obtained the death or censor date with the vital status current until May 30, 2022. We further identified the first occurrence of an inpatient admission for a primary diagnosis of MI, stroke or a major adverse limb event (MALE). MALE was defined as a composite of acute extremity ischemia or an emergency lower extremity vascular procedure. Inpatient admission information was available for all events that occurred in any VA medical center or a non-VA facility reimbursed by the VA. As such, the completeness of the follow-up data is near 100%.

Our coprimary end points for this study were major adverse cardiovascular event (MACE) defined as a composite of the first occurrence of MI, stroke, MALE, or death, and all-cause mortality. We also evaluated MI, stroke, and MALE as secondary end points.

### Statistical Analysis

We report baseline characteristics for the whole cohort and separately for each HOLC grade using count (frequencies), mean (SD) for categorical data, or median (IQR) for continuous data. We compared the baseline characteristics between HOLC grades using the χ^2^ test for categorical data or *t* test or Wilcoxon rank-sum test for continuous data after adjusting for multiplicity with the Bonferroni correction.

We used the Kaplan-Meier method to obtain the unadjusted cumulative incidence of MACE and all-cause mortality for the whole cohort. We also report the cumulative incidence of MACE for each HOLC grade and performed a pairwise comparison with the log-rank test (with HOLC grade A as the reference). For each nonfatal component of MACE, we obtained the event’s cumulative incidence in a competing risk framework with all-cause mortality as the competing event. We compared the incidence between HOLC grades using the Fine-Gray test.

For each studied end point (MACE and its individual components), to calculate the marginal risk for patients in the HOLC grade D category with HOLC grade A as reference, we built 3 Cox proportional hazards regression models as follows: model 1: unadjusted; model 2 (main model): adjusted for age, sex, and race and ethnicity; and model 3 (exploratory model): adjusted for age, sex, race and ethnicity, diabetes, chronic obstructive pulmonary disease, hypertension, atrial fibrillation, heart failure, baseline low-density lipoprotein cholesterol level, prior MI, prior percutaneous coronary intervention, obesity, chronic kidney disease, and the community deprivation index score^9^ (fit as a continuous variable). We present the results of these Cox proportional hazards regression models using hazard ratios (HRs) with 95% CIs.

To examine whether there was an association between HOLC grade and MI, stroke, and MALE, we fit competing risk models using the Fine-Gray method. We fit 3 incrementally adjusted models and present results using the subdistribution HR and the 95% CI. To evaluate heterogeneity, we tested for the possible association between HOLC groups and outcomes in subgroups of race and ethnicity and community deprivation index and report the *P* value for the interaction terms. To contextualize the absolute effect size of HOLC grade on outcomes, we performed restricted mean time lost for MACE by HOLC grade through 5 years of follow-up, adjusted for age, sex, and race and ethnicity (model 2). As patients were treated at 129 different VA medical centers, we included the center identifier as a random effect in our model using a γ distribution. Missing information in the covariates included in our fully adjusted model was minimal: baseline low-density lipoprotein cholesterol level, 7.49%; ethnicity, 3.29%; community deprivation index, 1.29%; and race, 1.05%. We used the multiple imputation method to develop 5 imputed data sets; fitting separate models for each data set we used the Rubin method to pool the summary effects from each model. All tests were 2 sided, and *P* < .05 was considered the statistically significant threshold. Data analysis was conducted with R, 3.6.3 (R Foundation for Statistical Computing).

To determine the mean time to MACE difference between HOLC grades, we first fit a parametric survival model (adjusted for age, sex, and self-reported race and ethnicity) with a Weibull distribution to our data. We then obtained, at regular time intervals, the difference in the restricted mean survival time between the HOLC grade A and D groups. This difference in restricted mean survival time is defined as the restricted mean time lost and describes the mean difference in the event occurrence between the 2 comparison groups. We calculated the 95% CI for the restricted mean survival time using the δ method.

## Results

**Figure 1.**
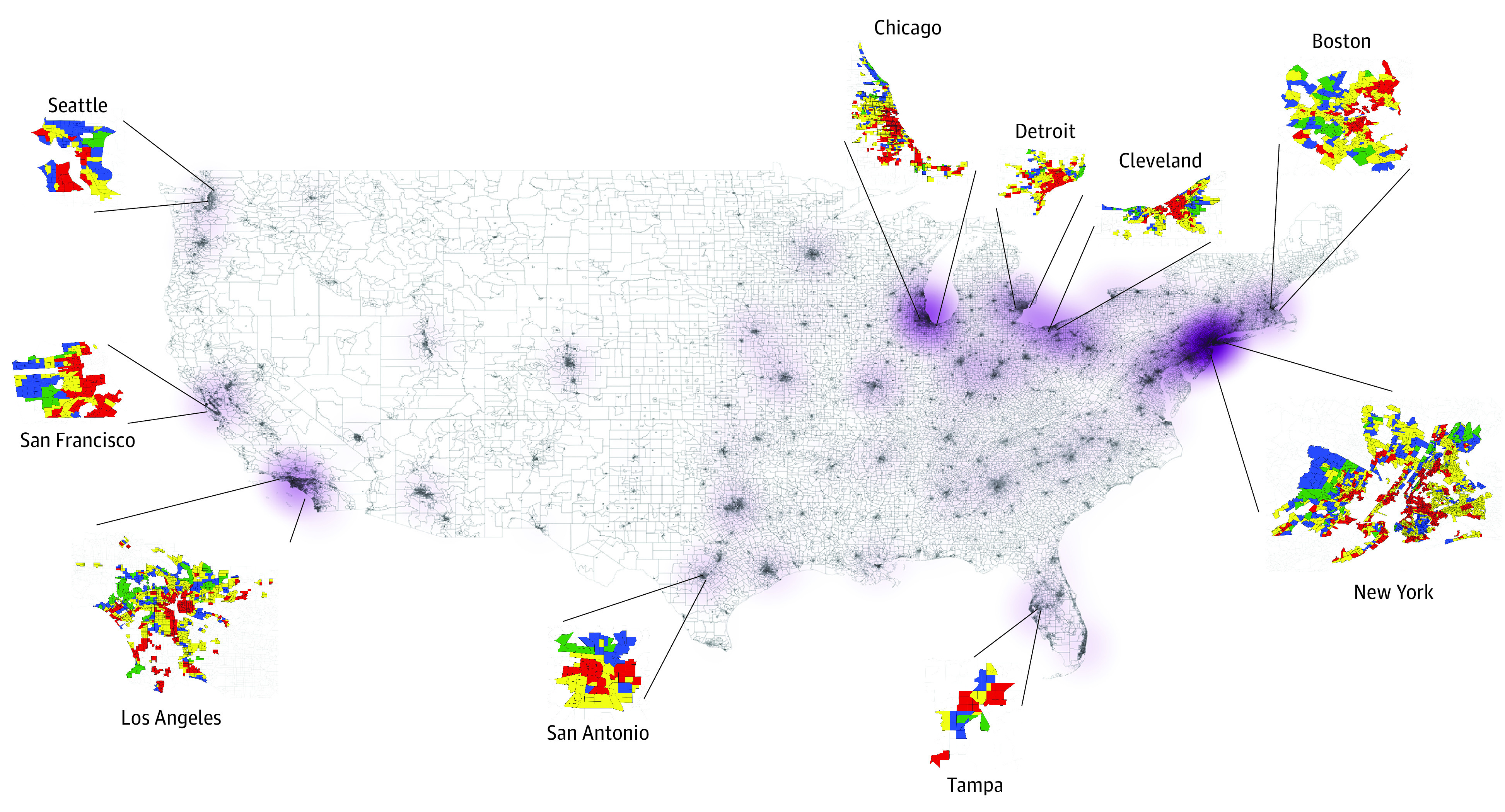
Veteran Cohorts Residing in Historically Home Owners’ Loan Corporation–Graded Neighborhoods Throughout the Continental US

A total of 79 997 patients with atherosclerotic vascular disease were included (eFigure 1 in Supplement 1). eTable 1 in Supplement 1 reports the characteristics of included and excluded patients. In the overall cohort, the mean (SD) age was 74.46 (10.16) years, 2281 (2.9%) were female, 77 716 (97.1%) were male, 44 584 (55.7%) were White, and 4341 (5.4%) were Hispanic. Our study included patients from 10 980 of the 13 480 census tracts (81.5%) graded by the HOLC. Overall, 7% of the patients resided in HOLC grade A neighborhoods, 20% in group B, 42% in group C, and 31% in grade D. The geographic distribution of these patients is shown in Figure 1.

Table 1 presents the baseline characteristics according to HOLC grade, with grades varied with respect to demographic composition. There were stepwise increments in the percentage composition of Asian (from 0.3% to 0.8%), Black (from 25.2% to 47.2%), and Hispanic (from 3.9% to 6.4%) populations across the HOLC spectrum (HOLC grades A to D) whereas there were stepwise decrements in the composition of the White population (from 68.2% [grade A] to 45.6% [grade D]).

**Table 1.** Baseline Characteristics of the Study Cohort by HOLC Grade

Characteristic	Overall	HOLC Grade				*P* value
		A	B	C	D	
No.	79 997	5204	16 383	33 952	24 458	
Condition, No. (%)						
Coronary artery disease	45 026 (56.3)	3234 (62.1)	9476 (57.8)	19 180 (56.5)	13 136 (53.7)	<.001
Cerebrovascular disease	18 043 (22.6)	1088 (20.9)	3599 (22.0)	7566 (22.3)	5790 (23.7)	
Peripheral artery disease	16 928 (21.2)	882 (16.9)	3308 (20.2)	7206 (21.2)	5532 (22.6)	
Age, mean (SD), y	74.46 (10.16)	76.58 (10.52)	75.20 (10.32)	74.48 (10.06)	73.48 (10.01)	<.001
Sex						.28
Male	77 716 (97.1)	5058 (97.2)	15 905 (97.1)	33 026 (97.3)	23 727 (97.0)	
Female	2281 (2.9)	146 (2.8)	478 (2.9)	926 (2.7)	731 (3.0)	
Race^a^						
American Indian or Alaska Native	914 (1.1)	68 (1.3)	173 (1.1)	365 (1.1)	308 (1.3)	<.001
Asian	463 (0.6)	18 (0.3)	81 (0.5)	166 (0.5)	198 (0.8)	
Black	29 873 (37.3)	1309 (25.2)	5418 (33.1)	11 602 (34.2)	11 544 (47.2)	
White	44 584 (55.7)	3550 (68.2)	9927 (60.6)	19 947 (58.8)	11 160 (45.6)	
Declined to report	3323 (4.2)	174 (3.3)	614 (3.7)	1505 (4.4)	1030 (4.2)	
Unknown	840 (1.1)	85 (1.6)	170 (1.0)	367 (1.1)	218 (0.9)	
Ethnicity^a^						
Hispanic	4341 (5.4)	201 (3.9)	713 (4.4)	1859 (5.5)	1568 (6.4)	<.001
Not Hispanic	73 474 (91.8)	4861 (93.4)	15 226 (92.9)	31 088 (91.6)	22 299 (91.2)	
Unknown	2182 (2.7)	142 (2.7)	444 (2.7)	1005 (3.0)	591 (2.4)	
Community deprivation index, mean (SD) (range: 0-1)	0.44 (0.11)	0.38 (0.11)	0.42 (0.11)	0.44 (0.11)	0.49 (0.11)	<.001
Smoking	23 887 (29.9)	1413 (27.2)	4683 (28.6)	10 182 (30.0)	7609 (31.1)	<.001
Chronic kidney disease	34 018 (42.5)	2092 (40.2)	6818 (41.6)	14 279 (42.1)	10 829 (44.3)	<.001
Obesity	24 815 (31.0)	1562 (30.0)	4968 (30.3)	10 627 (31.3)	7658 (31.3)	.04
Hypertension	66 435 (83.0)	4270 (82.1)	13592 (83.0)	28 175 (83.0)	20 398 (83.4)	.11
Diabetes	38 396 (48.0)	2310 (44.4)	7670 (46.8)	16 449 (48.4)	11 967 (48.9)	<.001
Heart failure	11 354 (14.2)	668 (12.8)	2203 (13.4)	4804 (14.1)	3679 (15.0)	<.001
Atrial fibrillation	13 254 (16.6)	1041 (20.0)	2883 (17.6)	5715 (16.8)	3615 (14.8)	<.001
Chronic obstructive pulmonary disease	16 817 (21.0)	993 (19.1)	3377 (20.6)	7198 (21.2)	5249 (21.5)	.001
Prior myocardial infarction	2427 (3.0)	162 (3.1)	532 (3.2)	1029 (3.0)	704 (2.9)	.20
Prior percutaneous coronary intervention	2110 (2.6)	149 (2.9)	495 (3.0)	908 (2.7)	558 (2.3)	<.001
Systolic blood pressure, mean (SD), mm Hg	134.81 (20.79)	133.92 (20.18)	134.53 (20.61)	134.79 (20.80)	135.20 (21.00)	<.001
Diastolic blood pressure, mean (SD), mm Hg	75.01 (11.93)	74.09 (11.99)	74.63 (11.95)	74.79 (11.82)	75.77 (12.02)	<.001
Low-density lipoprotein cholesterol, mean (SD), mg/dL	87.15 (34.56)	86.73 (33.57)	87.82 (34.66)	86.23 (34.18)	88.09 (35.18)	<.001

Abbreviation: HOLC, Home Owners’ Loan Corporation.

SI conversion factor: To convert cholesterol levels to millimoles per liter, multiply by 0.0259.

^a^ Race was self-reported and ethnicity was reported separately.

Across HOLC grades A through D, we observed an overall increase in the prevalence of cardiovascular disease risk factors, such as smoking (from 27.2% to 31.1%) and obesity (from 30.0% to 31.3%). Furthermore, compared with individuals who resided in HOLC grade A neighborhoods, those living in HOLC grade D neighborhoods had marginally higher low-density lipoprotein cholesterol levels, systolic blood pressure, and diastolic blood pressure (Table 1).

Patients residing in redlined areas were less likely than those residing in HOLC grade A areas to have atrial fibrillation (14.8% vs 20.0%), but more likely to have chronic kidney disease (44.3% vs 40.2%), heart failure (15.0% vs 12.8%), diabetes (48.9% vs 44.4%), chronic obstructive pulmonary disease (21.5% vs 19.1%), and a higher community deprivation index level (0.49 vs 0.38) (Table 1; eFigure 2 in Supplement 1).

**Figure 2.**
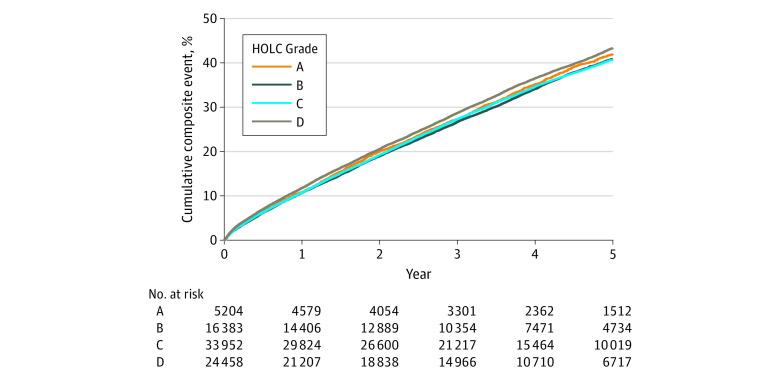
Cumulative Event Plots for Cardiovascular Outcomes Cumulative positive events for composite cardiovascular outcomes. HOLC indicates Home Owners’ Loan Corporation.

At a median follow-up of 4.0 (IQR, 2.6-5.3) years, 31 078 (38.84%) of the patients had composite cardiovascular outcomes (5589 had MI, 476 had stroke, 465 had vascular events, and 28 326 died). The cumulative rates of composite cardiovascular events were for 1 year, 11.04% (95% CI, 10.82%-11.26%); for 3 years, 23.17% (22.29%-23.47%); and for 5 years, 37.34% (36.97%-37.71%), and for all-cause mortality, cumulative rates were 8.37% (8.18%-8.56%) for 1 year, 23.17% (22.88%-23.47%) for 3 years, and 37.34% (36.97%o-37.71%) for 5 years (Figure 2).

**Figure 3.**
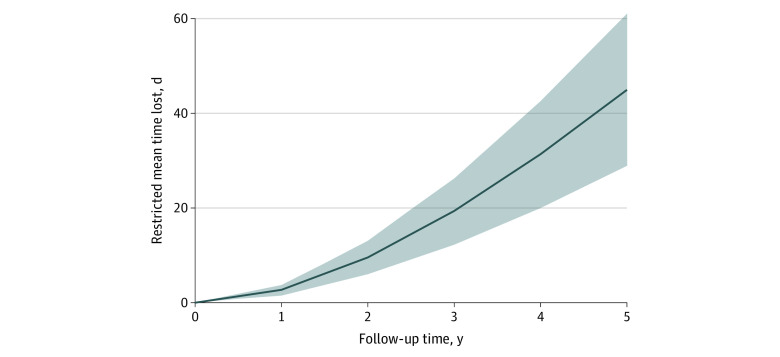
Restricted Mean Time Lost for Major Adverse Cardiovascular Events Days lost for major adverse cardiovascular events in Home Owners’ Loan Corporation grade D vs grade A neighborhoods through 5 years, adjusted for age, sex, and race and ethnicity (model 2). The shaded area indicates 95% CIs.

After adjustment for demographic factors in model 2 (age, sex, and race and ethnicity), patients residing in HOLC grade D neighborhoods, compared with those residing in HOLC grade A neighborhoods, had 14% higher hazards of MACE (HR, 1.139; 95% CI, 1.083-1.198; *P* < .001) and 13% higher hazards of all-cause mortality (HR, 1.129; 95% CI, 1.072-1.190; *P* < .001). After multivariable adjustment for demographic characteristics, social deprivation indices, and cardiovascular risk factors (model 3), compared with veterans residing in grade A neighborhoods, those residing in redlined (grade D) neighborhoods had increased risk of MACE (HR, 1.056; 1.004-1.110; *P* = .02) and all-cause mortality (HR, 1.055; 95% CI, 1.001-1.111; *P* = .02) (Table 2). Similar findings were noted when analysis of complete cases (without imputation) was performed (eTable 2 in Supplement 1). Considering HOLC as a continuous score, on adjusted analyses, we observed that every unit increase in the HOLC score was associated with an increased relative risk for MACE (HR, 1.016; 95% CI, 1.002-1.029; *P* = .01). Similarly, veterans residing in redlined neighborhoods had a 15% higher risk of MI (HR, 1.148; 95% CI, 1.011-1.303; *P* < .001) but not stroke (HR, 0.889; 95% CI, 0.584-1.353; *P* = .58) or MALE (eTable 3 in Supplement 1). During a 5-year follow-up period, the mean time to MACE was 45 days earlier in patients residing in HOLC grade D compared with HOLC grade A neighborhoods (Figure 3).

**Table 2.** Hazard Ratios With HOLC Grade and Major Adverse Cardiovascular Events and All-Cause Mortality

Variable	Model 1^a^		Model 2^b^		Model 3^c^	
	Hazard ratio (95% CI)	*P* value	Hazard ratio (95% CI)	*P* value	Hazard ratio (95% CI)	*P* value
**Major adverse cardiovascular event**						
HOLC A	1 [Reference]		1 [Reference]		1 [Reference]	
HOLC B	0.957 (0.909-1.007)	.09	1.002 (0.951-1.055)	.93	0.967 (0.917-1.020)	.22
HOLC C	0.976 (0.930-1.024)	.32	1.051 (1.001-1.104)	.04	0.991 (0.943-1.042)	.73
HOLC D	1.02 (0.978-1.080)	.26	1.139 (1.083-1.198)	<.001	1.056 (1.004-1.110)	.02
**All-cause mortality**						
HOLC A	1 [Reference]		1 [Reference]		1 [Reference]	
HOLC B	0.944 (0.895-0.995)	.03	1.001 (0.948-1.056)	.97	0.960 (0.913-1.011)	.63
HOLC C	0.954 (0.908-1.003)	.06	1.050 (0.998-1.105)	.06	0.977 (0.931-1.025)	.25
HOLC D	0.989 (0.940-1.040)	.67	1.129 (1.072-1.190)	<.001	1.055 (1.001-1.111)	.02

Abbreviation: HOLC, Home Owners’ Loan Corporation.

^a^ Model 1, unadjusted.

^b^ Model 2, adjusted for age, sex, race and ethnicity.

^c^ Model 3, adjusted for age, sex, race and ethnicity, diabetes, chronic obstructive pulmonary disease, hypertension, atrial fibrillation, heart failure, baseline low-density lipoprotein cholesterol level, prior myocardial infarction, prior percutaneous coronary intervention, obesity, chronic kidney disease, and the community deprivation index score.

The HR between HOLC grade and MACE was greater for patients of races other than White (HR, 1.154; 95% CI, 1.116-1.194) vs White (HR, 1.079; 95% CI, 1.041-1.117; *P* < .001 for interaction). Conversely, there were no significant differences between HOLC grade (D vs A, model 2) and MACE by ethnicity (Hispanic HR, 1.223; 95% CI, 1.103-1.357 vs non-Hispanic HR, 1.129; 1.102-1.158; *P* = .14 for interaction), and by community deprivation index (tertile 1: HR, 1.119; 95% CI, 1.065-1.176 vs tertile 2: HR, 1.090; 95% CI, 1.049-1.133 vs tertile 3: HR, 1.080; 95% CI, 1.036-1.127; *P* = .55 for interaction). eTable 4 in Supplement 1 shows that subgroup analysis according to race and ethnicity.

## Discussion

In this study, we sought to investigate neighborhood redlining and long-term outcomes among veterans with atherosclerotic cardiovascular disease and noted that living in historically redlined neighborhood was associated with a 13% higher risk for all-cause mortality, 14% higher risk of the composite MACE outcome, and 15% higher risk of MI. This risk was lower, yet remained significant, after adjusting for social vulnerability and comorbidity burden.

To our knowledge, few prior studies have linked neighborhood redlining and cardiovascular outcomes. Mujahid et al^4^ investigated 4779 participants in the Multi-Ethnic Study of Atherosclerosis residing in 600 census tracts and reported that redlining was linked with poorer cardiovascular health parameter score only among Black participants. A recent study by several authors of the presented study noted that, throughout the US, people living in redlined neighborhoods have a significantly higher prevalence of cardiovascular risk factors, coronary artery disease, stroke, and chronic kidney disease.^3^ This current study extends these findings to an individual-level longitudinal analysis of veterans at increased risk for events and noted that the association between neighborhood redlining and poor outcomes appears to be independent of traditional cardiovascular risk factors.

Redlining in our study continued to be linked with traditional cardiovascular risk factors. Veterans living in redlined neighborhoods had higher rates of hypertension, diabetes, obesity, smoking, and low-density lipoprotein cholesterol levels. These findings mirror those of earlier studies and suggest that at least part of the association between neighborhood redlining and cardiovascular outcomes is mediated by risk factors.^3,4^ Consistent with this hypothesis, the association between redlining and MACE and mortality was attenuated once risk factors were added to the model. Redlining may affect various social domains, which have been linked with poor cardiovascular health. For example, Corwin et al^10^ reported that various domains of adverse social determinants of health (including economic stability, neighborhood/built environment, education access, health care access, and social or community context) were associated with poor control of cardiovascular risk factors in patients with type 2 diabetes enrolled in the Health and Retirement Study. Future studies should incorporate individual social determinants of health and neighborhood social risk for comprehensive assessment of cardiovascular health.

The mechanisms of the observed associations remain speculative and likely multifactorial. For instance, earlier studies have suggested that years of inadequate investments in redlined neighborhoods have facilitated a confluence of a number of environmental exposures in the air, water, and soil resulting in protracted exposures^11^; poor neighborhood designs translating into reduced recreational land space, reduced availability of healthy foods, increased exposure to traffic and noise, and reduced tree cover have also been speculated to increase stress-related responses.^8,12,13^ For example, a recent study noted that redlining is associated with a wide array of present-day environmental exposures (eg, air pollution, light pollution, noise pollution, and less vegetation),^14^ which disproportionately affected racially minoritized populations.^11,15^ In a previous analysis, adjusting for environmental confounders such air pollution attenuated the association between redlining and cardiovascular outcomes (coronary artery disease, chronic kidney disease, and stroke), suggesting that air pollution may possibly play a role in the observed associations.^3^ Other intermediate outcomes of redlining include wealth inequality due to historical difficulty in obtaining loans and difficulty with access to credit contributing to wealth inequality, leading to increased financial insecurity and a lack of resources, which in turn can have negative effects on health. Limited educational opportunities in redlined areas may also contribute to poor health outcomes. For example, it has been reported that redlined areas have lower-performing schools,^16^ which can have long-term negative associations with future opportunities and overall health and well-being.

Earlier studies have documented that redlined neighborhoods still with the sequalae of structural racism may persistently confer inordinate stressor responses on the population living in these areas.^17,18,19^ Stress over time may be biologically transduced by amygdalar activity independently, resulting in arterial inflammation leading to MACE.^20^ Similarly, studies have noted that air pollution alone and in conjunction with, for instance, noise, can activate identical mechanisms and result in increased arterial inflammation.^21,22^ Furthermore, it is also postulated that the health consequences of racial discrimination and segregation can be persistent and intergenerational via epigenetic changes as an embodiment of racial inequalities.^23^ The combined effect of transgenerational social vulnerability and health consequences entailed by racial segregation create an unfavorable milieu for minoritized populations living in redlined neighborhoods.^24^ This further underscores the idea that one’s surrounding environment is a powerful estimator of health^25^ through mechanisms that are distinct and additive to traditional risk factors. Comprehensive characterization of the exposome, including the biologic, epigenetic, natural, and built environment, is therefore required to understand these relationships.

This study has implications for clinical practice and public health policy. First, this study builds on earlier work and suggests that historic sequelae of structural racism may be consistent factors in cardiovascular outcomes. Second, it highlights the need to consider neighborhood characteristics when assessing cardiovascular risk both for individuals and populations. Innovative strategies to improve cardiovascular health in redlined neighborhoods are urgently needed. These may include improving neighborhood designs by increasing greenspace,^8,26^ reducing access to tobacco via increasing taxes and elevating the legal age for purchasing,^27^ increasing access to healthy food,^28^ and implementing novel health care delivery models (eg, mobile health units)^29^ to improve cardiovascular outcomes.

### Limitations

These findings must be interpreted within the context of several limitations. First, this study investigated veterans, most of whom are men, and the findings therefore may not be generalizable to nonveteran populations. Second, HOLC graded only approximately 200 cities, and thus the final cohort size (79 997) is much smaller than the overall patient population (>1 million). Third, we did not account for changes in location. Fourth, this was a cohort of insured patients who are connected to health care services, which might reduce the effect size of redlining, especially as it relates to access to care and health insurance. Fifth, it is possible that residual confounding may have contributed to the observed findings that have small size of association, and we cannot exclude this possibility. Nevertheless, these findings in a veterans’ population that represents a large geographically and racially diverse contemporary cohort with atherosclerotic vascular disease with complete outcome ascertainment are unique advantages and sheds light on racist residential policies and environmental disinvestment in a high-risk population.

## Conclusions

In this cohort study of US veterans with atherosclerotic cardiovascular disease, those residing in historically redlined neighborhoods appear to continue to have a higher prevalence of traditional cardiovascular risk factors and higher risk of adverse events compared with those in other neighborhoods. Even nearly a century after its elimination, redlining is still adversely associated with cardiovascular events nationally.

## Notes

Supplement 1. **eFigure 1.** CONSORT Flow Diagram Presenting Cohort Selection **eFigure 2.** Distribution of the Community Deprivation Index Across HOLC Graded Census Tracts **eTable 1.** Baseline Characteristics Between Patients Included and Excluded From Our Cohort Study **eTable 2.** Complete Case Analysis **eTable 3.** The Association Between HOLC Grade and Cardiovascular Outcomes **eTable 4.** Subgroup Analysis of MACE According to Race and Ethnicity

Supplement 2.
Data Sharing Statement
