# Supplementary material for: Association Between Historical Neighborhood Redlining and Cardiovascular Outcomes Among US Veterans With Atherosclerotic Cardiovascular Diseases
Source: JAMA Netw Open. 2023 Jul 11;6(7):e2322727. doi: 10.1001/jamanetworkopen.2023.22727 (PMC10336624; doi:10.1001/jamanetworkopen.2023.22727)
Supplement: Supplement 1. — eFigure 1. CONSORT Flow Diagram Presenting Cohort Selection eFigure 2. Distribution of the Community Deprivation Index Across HOLC Graded Census Tracts eTable 1. Baseline Characteristics Between Patients Included and Excluded From Our Cohort Study eTable 2. Complete Case Analysis eTable 3. The Association Between HOLC Grade and Cardiovascular Outcomes eTable 4. Subgroup Analysis of MACE According to Race and Ethnicity [file jamanetwopen-e2322727-s001.pdf]

Supplementary Online Content

Deo SV, Motairek I, Nasir K, et al. Association between historical neighborhood redlining and cardiovascular outcomes among US veterans with atherosclerotic cardiovascular diseases. *JAMA Netw Open*. 2023;6(7):e2322727. doi:10.1001/jamanetworkopen.2023.22727

eFigure 1. CONSORT Flow Diagram Presenting Cohort Selection

eFigure 2. Distribution of the Community Deprivation Index Across HOLC Graded Census Tracts

eTable 1. Baseline Characteristics Between Patients Included and Excluded From Our Cohort Study

eTable 2. Complete Case Analysis

eTable 3. The Association Between HOLC Grade and Cardiovascular Outcomes

eTable 4. Subgroup Analysis of MACE According to Race and Ethnicity

This supplementary material has been provided by the authors to give readers additional information about their work.

© 2023 Deo SV et al. *JAMA Network Open.*

eFigure 1. CONSORT flow diagram presenting cohort selection.

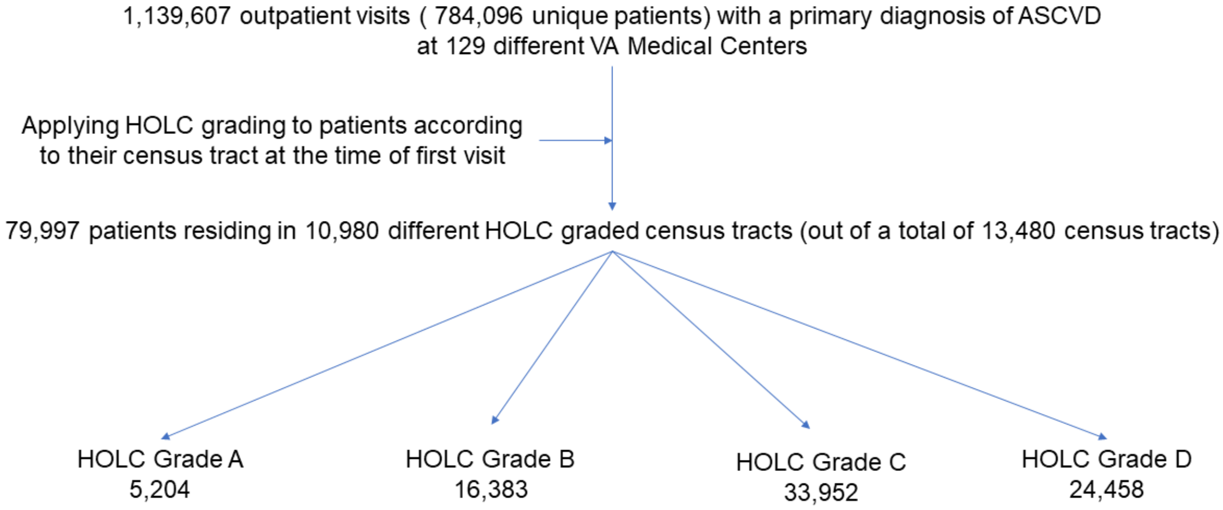

© 2023 Deo SV et al. *JAMA Network Open.*

eFigure 2. Distribution of the community deprivation index across HOLC graded census tracts

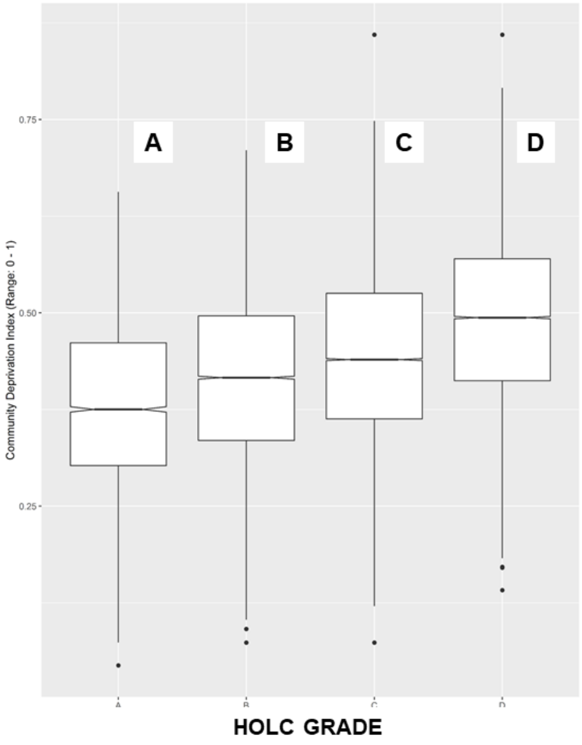

HOLC Grade	Median (IQR)
A	0.375 (0.302 , 0.461)
B	0.416 (0.335, 0.496)
C	0.440 (0.363, 0.525)
D	0.494 (0.412, 0.570)

IQR: interquartile range

HOLC=Home Owner's Loan Corporation; IQR = interquartile range

© 2023 Deo SV et al. *JAMA Network Open.*

eTable 1. Baseline Characteristics between patients included and excluded from our cohort study

	Included Patients (n = 79,997)	Excluded Patients (n = 704,099)
Age (years)	74.46 (10.16)	73.94 (9.89)
Female	2.9%	4%
Race		
Asian	0.6%	0.5%
Black	37.3%	8%
Declined	4.2%	4.3%
Native	1.1%	1.5%
White	55.7%	80.1%
Unknown	1.1%	1.8%
Ethnicity		
Hispanic	5.4%	4.6%
Not Hispanic	91.8%	91.8%
Unknown	2.7%	2.9%
Community deprivation index (Range: 0 – 1)	0.44 (0.11)	0.50 (0.26)
Hypertension	83%	83%
Diabetes	48%	47%

© 2023 Deo SV et al. *JAMA Network Open.*

Heart Failure	14.2%	12%
Atrial Fibrillation	16.6%	18.1%
Chronic Obstructive Pulmonary Disease	21%	21.2%
Prior Myocardial Infarction	3%	3.4%
Prior Percutaneous Coronary Intervention	2.6%	3%
Systolic Blood Pressure (mmHg)	134.81 (20.79)	133.40 (19.68)
Diastolic Blood Pressure (mmHg)	75.01 (11.93)	74.26 (11.12)
Low-Density Lipoprotein Cholesterol (mg/dL)	87.15 (34.56)	89.23 (38.01)

© 2023 Deo SV et al. *JAMA Network Open.*

eTable 2. Complete case analysis

As a sensitivity analysis, we also fit the fully adjusted model as a complete case analysis without imputation. We observed that this did not change our primary findings using the imputed data sets.

HOLC Grade (Ref: Grade A)	Hazard Ratio (95% Confidence interval)	p-value
B	0.96 (0.91, 1.01)	0.18
C	0.98 (0.93, 1.03)	0.50
D	1.06 (1.00, 1.12)	0.02

HOLC=Home Owner’s Loan Corporation

© 2023 Deo SV et al. *JAMA Network Open.*

eTable 3. The association between HOLC grade and cardiovascular outcomes

	Model 1	Model 2	Model 3
	Sub-distribution Hazard ratio (95% confidence interval)		
Myocardial Infarction (Ref. category HOLC-A)			
HOLC B	1.078 (0.950, 1.222), P=0.24	1.043 (0.918, 1.184), P=0.51	0.980 (0.861,1.116); p = 0.76
HOLC C	1.112 (0.987, 1.253), P=0.08	1.058 (0.937, 1.194), P=0.35	0.966 (0.853,1.093); p = 0.58
HOLC D	**1.310 (1.161, 1.478), P< 0.001**	**1.230 (1.088, 1.391), P< 0.001**	**1.148(1.011, 1.303); p = 0.03**
Stroke (Ref. category: HOLC-A)			
HOLC B	0.958 (0.623, 1.463), P=0.83	0.867 (0.564, 1.330), P=0.51	0.793 (0.523,1.221; p = 0.30
HOLC C	0.946 (0.630, 1.420), P=0.78	0.842 (0.560, 1.265), P=0.4	0.754 (0.505, 1.128); p = 0.17
HOLC D	1.089 (0.724, 1.638), P=0.68	0.952 (0.632, 1.434), P=0.81	0.889 (0.584, 1.353); p = 0.58
Major Adverse Limb Event (Ref. category: HOLC-A)			
HOLC B	1.013 (0.625, 1.640), P=0.95	0.921 (0.563, 1.502), P=0.74	Could not be reported as the regression model failed to converge
HOLC C	1.534 (0.982, 2.396), P=0.06	1.376 (0.872, 2.168), P=0.16	
HOLC D	1.534 (0.977, 2.408), P=0.06	1.328 (0.834, 2.113), P=0.23	

Model 1 = unadjusted; Model 2 = adjusted for age, sex, race, ethnicity; Model 3 = adjusted for age, sex, race, ethnicity, diabetes, COPD, hypertension, atrial fibrillation, heart failure, baseline LDL, prior MI, prior PCI, obesity, chronic kidney disease, and the community deprivation index

HOLC=Home Owner's Loan Corporation

© 2023 Deo SV et al. *JAMA Network Open.*

eTable 4. Subgroup analysis of MACE according to race and ethnicity

Subgroup	HOLC Grade	HR	p-value
White	B vs A	0.98 (0.92, 1.04)	0.61
	C vs A	0.98 (0.93, 1.05)	0.71
	D vs A	1.10 (1.03, 1.05)	0.003
Black	B vs A	0.91 (0.82, 1.00)	0.06
	C vs A	0.96 (0.87, 1.05)	0.40
	D vs A	1.00 (0.91, 1.09)	0.97
Hispanic	B vs A	0.83 (0.64, 1.09)	0.18
	C vs A	0.88 (0.69, 1.13)	0.33
	D vs A	1.01 (0.79, 1.29)	0.88
Not Hispanic	B vs A	0.97 (0.92, 1.02)	0.24
	C vs A	0.98 (0.93, 1.03)	0.49
	D vs A	1.05 (1.00, 1.10)	0.04

HOLC=Home Owner’s Loan Corporation; HR=hazard ratio

© 2023 Deo SV et al. *JAMA Network Open.*
