# Supplementary material for: Association Between Historical Neighborhood Redlining and Cardiovascular Outcomes Among US Veterans With Atherosclerotic Cardiovascular Diseases
Source: JAMA Netw Open. 2023 Jul 11;6(7):e2322727. doi: 10.1001/jamanetworkopen.2023.22727 (PMC10336624; doi:10.1001/jamanetworkopen.2023.22727)
Supplement: Supplement 2. — Data Sharing Statement [file jamanetwopen-e2322727-s002.pdf]

Data Sharing Statement

Deo. Association Between Historical Neighborhood Redlining and Cardiovascular Outcomes Among US Veterans With Atherosclerotic Cardiovascular Diseases. *JAMA Netw Open*. Published July 11, 2023. doi:10.1001/jamanetworkopen.2023.22727

Data
Data available: No

Additional Information
Explanation for why data not available: This study used VA data, which are restricted from being shared with outside investigatory by policy
